# A review of instrumental variable estimators for Mendelian randomization

**DOI:** 10.1177/0962280215597579

**Published:** 2015-08-17

**Authors:** Stephen Burgess, Dylan S Small, Simon G Thompson

**Affiliations:** 1 Department of Public Health and Primary Care, University of Cambridge, Cambridge, UK; 2 Department of Statistics, The Wharton School, University of Pennsylvania, PA, USA

**Keywords:** Instrumental variable, comparison of methods, causal inference, weak instruments, finite-sample bias, Mendelian randomization

## Abstract

Instrumental variable analysis is an approach for obtaining causal inferences on the effect of an exposure (risk factor) on an outcome from observational data. It has gained in popularity over the past decade with the use of genetic variants as instrumental variables, known as Mendelian randomization. An instrumental variable is associated with the exposure, but not associated with any confounder of the exposure–outcome association, nor is there any causal pathway from the instrumental variable to the outcome other than via the exposure. Under the assumption that a single instrumental variable or a set of instrumental variables for the exposure is available, the causal effect of the exposure on the outcome can be estimated. There are several methods available for instrumental variable estimation; we consider the ratio method, two-stage methods, likelihood-based methods, and semi-parametric methods. Techniques for obtaining statistical inferences and confidence intervals are presented. The statistical properties of estimates from these methods are compared, and practical advice is given about choosing a suitable analysis method. In particular, bias and coverage properties of estimators are considered, especially with weak instruments. Settings particularly relevant to Mendelian randomization are prioritized in the paper, notably the scenario of a continuous exposure and a continuous or binary outcome.

## 1 Introduction

Instrumental variable analysis is the exploitation of a natural experiment to obtain causal inferences in an observational setting. An instrumental variable is a factor that is correlated with the exposure (usually a putative causal risk factor), but is not associated with any confounder of the exposure–outcome association, nor is there any pathway by which the instrumental variable can influence the outcome other than via the exposure of interest.^1,2^ Usually, an instrumental variable is in some way external to the relationship between the exposure and outcome and formally must be exogenous to the statistical model relating the exposure to the outcome (that is, not associated with the error term in the equation).^3^ A natural experiment is analogous to a randomized trial, except that the value of the exposure is influenced by nature rather than by an investigator on the basis of randomized allocation.^4^

A source of potential instrumental variables is genetic variants.^5^ A genetic variant is a section of genetic code that differs between individuals. Genetic variants are good candidate instrumental variables: the function of many genes is known and well characterized; genetic variants are fixed at conception, and so do not change due to environmental factors, thus avoiding reverse causation; and genetic variants are generally inherited independently, meaning that they tend to be specific in their associations. The use of genetic variants as instrumental variables in observational data has been termed ‘Mendelian randomization’.^6,7^

Many reviews exist on the use of instrumental variables, in particular on the assumptions necessary to be an instrumental variable,^8,9^ assessing the validity of instrumental variables,^10^ assumptions necessary for estimation of a causal effect parameter using instrumental variables,^11–13^ methods for effect estimation with multiple instrumental variables,^14^ with binary outcomes,^15,16^ methods for the estimation of odds ratios,^17^ and guidelines for the reporting of instrumental variable analysis.^18^ We seek to complement this literature by contributing a review to compare methods for instrumental variable analysis, with accompanying practical guidelines on their use. This review will focus on methodological issues that are particularly relevant to Mendelian randomization: the scenario of a continuous exposure and a continuous or binary outcome; statistical inference and the construction of confidence intervals; and the use of multiple instruments, in particular multiple weak instruments. A ‘weak instrument’ is an instrumental variable that explains a relatively small proportion of variance in the exposure.^19^ Although these are issues particularly germane to Mendelian randomization, they are also relevant to instrumental variable analyses in other contexts.

After framing the typical context of a Mendelian randomization investigation that we assume to hold (Section 2), we proceed to consider in turn the ratio of coefficients method (Section 3), two-stage methods (Section 4), likelihood-based methods (Section 5), and semi-parametric methods (Section 6). This order corresponds roughly to the complexity of the methods, with the simplest ones first. This is not an exhaustive list of methods that have been proposed for instrumental variable analysis, but it covers a wide range of commonly used methods. The methods are contrasted in terms of bias, coverage, efficiency, robustness to misspecification, and the existence of finite moments. We outline the problem of weak instruments and present a comparison of estimates from methods with weak instruments (Section 7). We also introduce a score-based (or ‘allele score’) approach to instrumental variable estimation, conceived for use with multiple weak instruments. We conclude by discussing methodological issues relating to the efficiency and validity of instrumental variable analyses, and to the use of instrumental variable methods in practice (Section 8).

## 2 Context of the problem

We assume that individual-level data are available from a single data source on an exposure *X*, an outcome *Y*, and an instrumental variable (IV) *Z*, or set of IVs $Z_{1}$, $Z_{2}$, …, $Z_{K}$. Usually the outcome is disease, although there is no methodological restriction as to what outcomes can be considered. The exposure is a putative causal risk factor for the outcome and is assumed to be continuous. A binary exposure is common for IV estimation in the context of a randomized trial to estimate the causal effect of treatment, where the IV is randomization.^20^ In this case, randomization typically has a large effect on the treatment received for a subgroup of individuals known as the compliers.^21^ However, in Mendelian randomization, the exposure is almost always continuous, and genetic variants typically have small effects on the exposure that are thought to affect all individuals in the population. Although it is possible to consider a dichotomized risk factor as an exposure (such as presence of hypertension in the case of blood pressure), a valid IV for the continuous exposure is not generally a valid IV for the dichotomized exposure, as there is an alternative causal pathway from the IV to the outcome via the true value of the continuous exposure.^10^

### 2.1 Genetic variants used in Mendelian randomization

We here provide a simplistic introduction to genetics with the aim of explaining the format of genetic data used in applied Mendelian randomization investigations; more detailed information on genetics for Mendelian randomization can be found in other papers.^5,6,22^

Genetic variants used as IVs in Mendelian randomization are usually single-nucleotide polymorphisms (SNPs). A SNP is a variation at a single position of the DNA sequence in some individuals in a population, where the DNA sequence has one letter replaced with another letter. The different possible letters that may appear at this position are known as alleles. Most SNPs are diallelic, having two alleles: one that is more common, known as the wild-type or major allele, and the other known as the variant or minor allele. For each individual, a SNP can be represented as a random variable by the number of variant alleles; this can take the values 0, 1, or 2, because each individual has two copies of the DNA sequence (two chromosomes). In some cases, the SNP may be regarded as a dichotomous variable, taking values 0 and 1: either for statistical reasons, if the variant allele is rare so that only a very small number of individuals in the sample have two copies, or for biological reasons, if individuals with a single copy of the variant allele have similar average values of the exposure as those with zero copies (recessive model) or as those with two copies (dominant model). Alternatively, the IV may be a copy number variant (a repeated section of the DNA code where the number of copies of the section varies between individuals),^23^ or an allele score (Section 7.3); in these cases, the IV would be a discrete variable taking positive values or a continuous variable.

### 2.2 Causal estimand

It has been established that the core IV assumptions only identify bounds for the causal effect rather than a single causal estimate.^24,25^ However, these bounds are extremely imprecise and even in large samples are rarely informative. We do not recommend their use in practical Mendelian randomization investigations. We here assume that the IV assumptions are satisfied; otherwise, even these bounds can be biased and highly misleading, particularly if the IVs are weak.^26^

With a binary exposure and a single IV, under the assumption of monotonicity in the effect of the IV on the exposure, the causal estimand from an IV analysis is known as a local average treatment effect^27^ or (in particular in the case of a binary IV) a complier-averaged causal effect.^28^ It represents the average change in the outcome for a change in the exposure amongst those individuals whose value of the exposure is affected by their value of the IV.^11^ Monotonicity means that the exposure for each individual would be increased (or alternatively for each individual would be decreased) or unchanged if that person had $Z=z_{1}$ compared to if they had $Z=z_{0}$ for all $z_{1}>z_{0}$. In the language of principal stratification for randomized trials, this is equivalent to the assumption that there are no defiers.^21^ For genetic variants that have proven biological links with the risk factor, the monotonicity assumption is plausible, as such biological effects seem likely to act in the same direction across all individuals. However, the monotonicity assumption is unlikely to hold if the IV is an allele score (Section 7.3), particularly if it is an unweighted or imprecisely weighted score. In any case, the monotonicity assumption is not testable from empirical data. If the monotonicity assumption is not satisfied, then the causal effect of the exposure on the outcome can be estimated consistently if it is constant for all individuals across the population.^29^ A weaker version of this assumption, that of no modification of the causal effect across levels of the instrument within the treated, can be used to estimate the effect of treatment in the treated.^30^

With a continuous exposure, to identify a causal parameter, it is necessary to make some parametric assumptions, for example homogeneity and linearity in the causal exposure–outcome association with no effect modification.^9,12^ The causal estimand is then the average change in the outcome for a unit change in the exposure. If there are multiple IVs, it is convenient to assume that the causal effect of the exposure on the outcome is homogeneous, so that all the IVs identify the same causal quantity. However, this assumption is questionable, particularly if the genetic variants used as IVs affect different biological pathways and so affect the value of the exposure in different ways. This assumption is best addressed by a sensitivity analysis, comparing IV estimates based on different subsets of IVs, particularly if the subsets correspond to groups of genetic variants with distinct biological functions, and can be tested formally by an overidentification test (Section 8.3).

With a binary outcome, it is common in conventional observational studies to express the association of interest as a relative risk or an odds ratio, rather than as an average change in the outcome (which would represent an effect on the risk difference scale). This is problematic in the case of an odds ratio, as the odds ratio is a non-collapsibility measure of association, meaning that its value depends on the distribution of risk factors in the population that affect the outcome even if these variables are not confounders of the relationship between the exposure and outcome.^31^ It is therefore recommended that odds ratios should only be estimated in a case–control setting, as other measures of association are sensitive to case–control sampling. We return to the problem of defining and estimating a causal odds ratio in the context of the individual IV methods.

In this paper, we presume that a causal effect estimate is the desired outcome of a Mendelian randomization analysis. In many cases, testing for an association between an IV and the outcome, to assess the causal role of the exposure and the direction of the causal effect without estimating a causal parameter, is sufficient.^32^ Such a test can be performed by a variety of methods, but most commonly using linear regression with a continuous outcome and using linear or logistic regression with a binary outcome. Although a causal estimate is likely to differ from the effect of a clinical intervention on the same exposure in practice,^7^ an effect estimate may be desired, for instance, to give a quantitative indication of the importance of the exposure in relation to the outcome. We proceed to consider various methods for IV estimation in the context described above; however, deviations in many of these features (such as the availability of individual-level data, linearity of the causal effect, homogeneity of the IV estimate from different IVs) are addressed throughout the paper and in particular in the discussion.

## 3 Ratio of coefficients method

The ratio of coefficients method, or the Wald method,^33^ is the simplest way of estimating the causal effect of the exposure (*X*) on the outcome (*Y*). The ratio method uses a single IV (*Z*); all other methods in this paper are able to include data on multiple IVs.

### 3.1 Continuous outcome

If the coefficient of the IV in the regression of the exposure on the IV is written as β^X|Z and the coefficient of the IV in the regression of the outcome on the IV is written as β^Y|Z, then the ratio estimate of the causal effect is

(1) Ratiomethodestimate=β^Y|Zβ^X|Z

Intuitively, we can think of the ratio method as saying that the change in the outcome for a unit increase in the exposure is equal to the change in the exposure for a unit increase in the IV, scaled by the change in the exposure for a unit increase in the IV. The causal estimate is usually expressed as the change in the outcome resulting from a unit change in the exposure, although changes in the outcome corresponding to different magnitudes of change in the exposure could be quoted instead.

If the IV is dichotomous, taking values 0 and 1, then the ratio estimate simplifies to

(2) $$\text{Ratiomethodestimate}(\text{dichotomousIV})=\frac{{\bar{y}}_{1}-{\bar{y}}_{0}}{{\bar{x}}_{1}-{\bar{x}}_{0}}$$

where ${\bar{y}}_{1}$ is the average value of the outcome amongst those with an IV value of 1, and so on. If the effect of the exposure on the outcome is assumed to be linear, then the ratio estimate is the causal effect on the outcome of an exposure of *x*+1 units versus an exposure of *x* units. If the effect is not linear, then theoretical and empirical considerations have demonstrated that the ratio estimate approximates an average causal effect of a population intervention in the exposure, for the population under analysis, with the assumption that the effect of the IV on the exposure is constant in the population^34^ (Section 8.1).

We note that the ratio estimate can be calculated simply from the coefficients β^X|Z and β^Y|Z and as such only requires the availability of summarized data, not individual-level data, from the population. We return to consider IV estimation using summarized data in more detail in Section 8.6.

### 3.2 Binary outcome

Generally in epidemiological applications, disease is the outcome of interest. Disease outcomes are often dichotomous. We use the epidemiological terminology of referring to an individual with an outcome event as a case (*Y*=1), and an individual with no event as a control (*Y*=0).

With a binary outcome, the coefficient β^Y|Z in the ratio estimate (equation (1)) is again taken from regression of the outcome on the IV. The regression model used could in principle be linear, so that the IV estimate represents the change in the probability of an event for a unit change in the exposure. However, with a dichotomous outcome, log-linear or logistic regression models are generally preferred by epidemiologists, where the IV estimate represents the log relative risk or log odds ratio, respectively, for a unit change in the exposure. In a case–control setting, measures based on risk differences depend on the choice of the ratio of cases to controls, whereas odds ratios are invariant to outcome-dependent sampling. More generally, relative risks and odds ratios (which approximate relative risks under the rare disease assumption^35^) are based on multiplicative risk models, which may be more plausible than additive risk models, and are easier to communicate.^36^

With a dichotomous IV, the ratio estimate simplifies as with a continuous outcome

(3) $$\text{Ratiomethodlogrelativerisk}(\text{orlogoddsratio})\text{estimate}(\text{dichotomousIV})=\frac{{\bar{y}}_{1}-{\bar{y}}_{0}}{{\bar{x}}_{1}-{\bar{x}}_{0}}$$

where ${\bar{y}}_{j}$ is the log of the probability of an event, or the log odds of an event, in IV subgroup *j*=0,1.

If the structural model relating the log of the probability of the outcome to the exposure is linear in the exposure with no effect modification, then the ratio estimate calculated using log-linear regression will be consistent for the causal log relative risk.^12^ The analogous consistency property does not hold in the case of a logistic-linear relationship between the probability of the outcome and the exposure, as the odds ratio is not a collapsible measure of association (see Section 4.2).

### 3.3 Case–control data

When case–control data are available, it is usual to make inferences on the IV-exposure association using only the controls.^37^ This makes the assumption that the association of the IV with the exposure in the controls is similar to that in the general population, which is true for a rare disease.^38^ This is necessary for two reasons. The first is reverse causation, as measurements of the exposure in a retrospective setting may be distorted by a disease event. Second, over-recruitment of cases into the study means that the distribution of confounders in the case–control sample is different to that in the general population. An association may be induced between the IV and the confounders, leading to possible bias in the IV estimate.^9^ This affects not only the ratio method but all IV methods. The IV–outcome association estimate should not be affected provided that logistic regression is used, as logistic regression estimates are invariant to outcome-dependent sampling. It is also necessary to assume that the probability of recruitment into the study is not dependent on the genetic variants used as IVs. For example, a genetic variant associated with increased mortality after an outcome event would appear to be protective rather than deleterious if the individuals who died were excluded from the study.

If the outcome is common and its prevalence in the population from which the case–control sample was taken is known, such as in a nested case–control study, then inferences on the IV–exposure association can be obtained using both cases and controls, provided that measurements of the exposure in cases were taken prior to the outcome event. This analysis can be performed by weighting the sample so that the proportions of cases and controls in the reweighted sample match those in the underlying population.^38^

### 3.4 Confidence intervals

Confidence intervals for the ratio estimate can be calculated in several ways.

#### 3.4.1 Normal approximation

The simplest way is to use a normal approximation. With a continuous outcome, standard errors and confidence intervals can be taken from the two-stage least squares method (see Section 4). Alternatively, the following approximation can be used, based on the first terms of the delta method expansion for the variance of a ratio

(4) Asymptotic standarderror of ratio estimate=se(β^Y|Z)2β^X|Z2+β^Y|Z2se(β^X|Z)2β^X|Z4-2β^Y|Zcov(β^X|Z,β^Y|Z)β^X|Z3

If the numerator and denominator of the ratio estimator are uncorrelated, then the third term can be omitted; in any event, such correlation is unlikely to have a considerable impact on confidence intervals.^39^

However, asymptotic normal approximations for the IV estimate may result in overly narrow confidence intervals, especially if the sample size is not large or the IV is weak (Section 7). This is because IV estimates are not normally distributed.^19^

#### 3.4.2 Fieller’s theorem

Alternatively, if the regression coefficients in the ratio method β^Y|Z and β^X|Z are assumed to be normally distributed, critical values and confidence intervals for the ratio estimator may be calculated using Fieller’s theorem.^5,40^ Fieller’s theorem gives confidence intervals that have one of three possible forms^41^:

- The interval may be a closed interval [*a*,*b*],
- The interval may be the complement of a closed interval (−∞,*b*]∪[*a*,∞), and
- The interval may be unbounded.

where *a* and *b* are functions of the association estimates β^Y|Z and β^X|Z and their standard errors. Confidence intervals from Fieller’s theorem may be preferred to those from an asymptotic normal approximation when the IV is weak.^42^ A tool to calculate confidence intervals from Fieller’s theorem is available online (https://sb452.shinyapps.io/fieller).

#### 3.4.3 Bootstrapping

As an alternative approach, also applicable to any of the following methods, confidence intervals can be calculated by bootstrapping.^43^ The simplest way of constructing a bootstrapped confidence interval is by taking several random samples with replacement from the data of the same sample size. The empirical distribution of the IV estimator in the bootstrapped samples approximates the true distribution of the IV estimator.^44^ However, there are some concerns about the behaviour of bootstrapped confidence intervals with weak instruments.^45^

#### 3.4.4 Other approaches

Alternative approaches for inference with weak instruments not discussed further here are confidence intervals based on inverting a test statistic, such as the Anderson–Rubin test statistic^46^ or the conditional likelihood ratio test statistic.^47^ A variance estimator for a generalized empirical likelihood approach with good coverage properties has also been developed.^48^ These intervals give appropriate confidence levels under the null hypothesis with weak instruments but may be underpowered with stronger instruments. However, unlike Fieller’s theorem, Anderson–Rubin and conditional likelihood ratio test confidence intervals can be calculated based on multiple IVs. These methods have been discussed in detail elsewhere^49,50^ and implemented in Stata^51^ and R.^52^ We recommend the Fieller’s theorem, Anderson–Rubin, or conditional likelihood ratio approaches for obtaining causal inferences and confidence intervals in practice with a weak instrument.

### 3.5 Absence of finite moments

One peculiar property of the ratio estimator is that its mean is not finite.^53,54^ This implies that, if you generated data on the exposure and outcome from a model with a valid IV and calculated the ratio estimate a large number of times, the mean value of these ratio estimates would become arbitrarily high (or low). This is due to the fact that there is a finite probability that the denominator in the ratio estimate (β^X|Z) is very close to zero, leading to a large IV estimate. In practice, this is unlikely to be a serious issue since, if β^X|Z were close to zero, the IV would be considered invalid as the assumption that the IV is associated with the exposure would appear to be violated. Theoretically, the absence of a finite mean makes comparison of IV methods more difficult, as the (mean) bias of the ratio estimate, defined as the difference between the mean of the IV estimator over its distribution and the true value of the causal effect, cannot be calculated. We therefore additionally consider the median bias, the difference between the median of the IV estimator and the true causal effect, when comparing different methods for IV estimation.^19^

## 4 Two-stage methods

A two-stage method comprises two regression stages: the first-stage regression of the exposure on the IVs, and the second-stage regression of the outcome on the fitted values of the exposure from the first stage. Both two-stage and likelihood-based methods (Section 5) make parametric assumptions for the identification of causal effects; this is contrasted with the weaker semi-parametric associations of the methods introduced in Section 6.

### 4.1 Continuous outcome – Two-stage least squares

With continuous outcomes and a linear model, the two-stage method is known as two-stage least squares (2SLS). It can be used with multiple IVs. In the first-stage regression, the exposure (*X*) is regressed on the IV(s) (*Z*) to give fitted values of the exposure (X^|Z). In the second-stage regression, the outcome (*Y*) is regressed on the fitted values for the exposure (X^|Z) from the first-stage regression. The causal estimate is this second-stage regression coefficient for the change in the outcome caused by a unit change in the exposure.

With a single IV, the 2SLS estimate is the same as the ratio estimate. With multiple IVs, the 2SLS estimator may be viewed as a weighted average of the ratio estimates calculated using the instruments one at the time, where the weights are determined by the relative strengths of the instruments in the first-stage regression.^55^

Suppose we have *K* instrumental variables available. With data on individuals indexed by *i* = 1, … , *N* who have exposure $x_{i}$, outcome $y_{i}$ and assuming an additive linear model for the IVs $z_{\mathrm{ik}}$ indexed by *k* = 1, … , *K*, the first-stage regression model is

(5) $$x_{i}=\alpha_{0}+\sum_{k}\alpha_{k}z_{\mathrm{ik}}+{ɛ}_{\mathrm{Xi}}$$

The fitted values x^i=α^0+∑kα^kzik are then used in the second-stage regression model

(6) yi=β0+β1x^i+ɛYi

where ${ɛ}_{\mathrm{Xi}}$ and ${ɛ}_{\mathrm{Yi}}$ are independent error terms. The causal parameter of interest is $\beta_{1}$. If both models are estimated by standard least-squares regression, both the error terms are implicitly assumed to be homoscedastic and normally distributed.

Although estimation of the causal effect in two stages (a sequential regression method) gives the correct point estimate, the standard error from the second-stage regression (equation (6)) is not correct.^56(p. 188)^ This is because it does not take into account the uncertainty in the first-stage regression. Under homoscedasticity of the error term in the equation

(7) $$y_{i}=\beta_{0}+\beta_{1}x_{i}+ɛ{'}_{\mathrm{Yi}}$$

the asymptotic variance of the 2SLS estimator is

(8) σ^2(XTZ(ZTZ)-1ZTX)-1=σ^2(X^TX^)-1

where σ^2 is an estimate of the variance of the residuals from equation (7), and *Z* is a *N* by *K* + 1 matrix of the IVs (including a constant term) and *X* is a *N* by 2 matrix of the risk factor and a constant term. Robust standard errors are often used in practice, as estimates are sensitive to heteroscedasticity and misspecification of the equations in the second-stage model. However, in the continuous outcome case, consistent estimates are obtained even when the first-stage model is misspecified.^57^

When all the associations are linear and the error terms normally distributed, the 2SLS estimator has a finite *k*th moment when there are at least (*k*+1) IVs.^53^ Therefore, the mean of a 2SLS estimator is only defined when there are at least 2 IVs, and the variance is only defined when there are at least 3 IVs. Unlike in an observational analysis, in which estimates from conventional regression (ordinary least squares) are attenuated by measurement error in the exposure,^58^ the bias of effect estimates from the 2SLS method (and consequently from the ratio method amongst others) is not affected by classical (non-differential) measurement error in the exposure.^59^

### 4.2 Binary outcome

The analogue of 2SLS with binary outcomes is a two-stage estimator where the second-stage (exposure–outcome) regression uses a log-linear or logistic regression model. These methods are implemented in order to estimate a causal relative risk or odds ratio parameter. They can be implemented using a sequential regression method by performing the two regression stages in turn. Estimates from such an approach will be overly precise, as uncertainty in the first-stage regression is not accounted for; however, this over-precision may be slight if the standard error in the first-stage coefficients is low. This can be resolved by the use of a likelihood-based method (Section 5.3) or a bootstrap method.^60^

As with the ratio IV estimator, in a case–control study, it is important to undertake the first-stage regression only in the controls, not the cases (Section 3.3). Fitted exposure values for the cases are obtained by substituting their IV values into the first-stage regression model.

Two-stage regression methods with non-linear second-stage regression models (such as logistic regression) have been criticized and called ‘forbidden regressions’.^56(p. 190)^ This is because the non-linear model does not guarantee that the residuals from the second-stage regression are uncorrelated with the instruments.^61^ There is current debate about the interpretation and validity of such estimates, especially when the measure of association is non-collapsible.

A measure of association, such as an odds ratio or relative risk, is collapsible if, when it is constant across the strata of the covariate, this constant value equals the value obtained from the overall (marginal) analysis. Non-collapsibility is the violation of this property.^62^ The relative risk and absolute risk difference are collapsible measures of association. Odds ratios are generally non-collapsible.^63^ An odds ratio estimated in an observational study by conventional multivariable logistic regression is conditional on those covariates adjusted for in the analysis. Unless adjustment is made (Section 8.2), an odds ratio estimated in an IV analysis is marginal on these covariates. The odds ratio from a ratio or two-stage analysis method is conditional on the IV, but marginal in all other variables, including the exposure itself if it is continuous.^64^ This approximates a population-averaged odds ratio from a randomized trial, where the intervention is to increase the exposure by one unit in all individuals across the population.^42^ A population-averaged odds ratio can be calculated as the average odds in the population for one value of the exposure distribution divided by the average odds for another value of the exposure distribution – for instance, the distribution of the exposure in the population increased uniformly by one unit, and the original distribution of the exposure in the population.

Despite the consequences of non-collapsibility, the two-stage estimator with a logistic second-stage model still provides a valid test of the null hypothesis.^17^

### 4.3 Adjusted two-stage method

An adjusted two-stage method has been proposed, where the residuals from the first-stage regression are included in the second-stage regression. This has been referred to as a control function approach,^65^ or two-stage residual inclusion method.^66^ If we have a first-stage regression of *X* on *Z* with fitted values X^|Z and residuals R^|Z=X-X^|Z, then the adjusted two-stage estimate comes from a second-stage regression additively on X^|Z and R^|Z (or equivalently on *X* and R^|Z). The residuals from the first-stage regression incorporate information on confounders.

If the outcome is continuous, then inclusion of these residuals in a second-stage linear regression model does not change the estimate, as the residuals are orthogonal to the fitted values. If the outcome is binary, inclusion of these residuals in a second-stage logistic regression model implies that the IV estimate is conditional on these residuals. Numerically, it brings the IV estimate closer to the subject-specific log odds ratio; the parameter $\beta_{1}$ in a logistic-linear model^67^

(9) $$\text{logitP}(Y=1|X=x,U=u)=\beta_{0}+\beta_{1}x+\beta_{2}u$$

where *U* represents all other covariates (whether confounders or not) in the probability model. Some investigators have therefore recommended the adjusted two-stage method when the second-stage regression is logistic on the premise that it is less biased than the unadjusted two-stage method.^66^

Under a particular choice of mathematical model in which the first-stage residual is equal (up to a multiplicative constant) to the term $\beta_{2}u$ in equation (9), the adjusted two-stage estimate is consistent for the parameter $\beta_{1}$.^66^ However, this mathematical model is unrealistic, and in general the adjusted two-stage estimate is biased for this parameter.^15,68^ Further, when the covariates are unknown, as is usual in an IV analysis, it is not clear what variable is represented by the first-stage residuals, and so which covariates the adjusted two-stage estimate is conditional on and which it is marginal across. It is uncertain what odds ratio is being estimated by an adjusted two-stage approach, that is, to what question is the adjusted two-stage estimate the answer. We therefore do not recommend adjustment for the first-stage residuals in a two-stage method.

## 5 Likelihood-based methods

The above methods are not likelihood based and do not provide maximum likelihood estimates, which have the desirable properties of asymptotic unbiasedness, normality, and efficiency. So we next consider likelihood-based methods.

### 5.1 Limited information maximum likelihood

If each individual *i* = 1, … ,  *N* has exposure $x_{i}$, outcome $y_{i}$, and IVs $z_{\mathrm{ik}}$ indexed by *k* = 1, … , *K*, we can assume the following model

(10) $$x_{i}=\alpha_{0}+\sum_{k}\alpha_{k}z_{\mathrm{ik}}+{ɛ}_{\mathrm{Xi}}$$

$$y_{i}=\beta_{0}+\beta_{1}x_{i}+{ɛ}_{\mathrm{Yi}}$$

where the two error terms have a bivariate normal distribution.^46^ (These error terms differ from those defined in equations (5) and (6).) The causal parameter of interest is $\beta_{1}$. Correlation between ${ɛ}_{X}$ and ${ɛ}_{Y}$ is due to confounding. We can calculate the maximum likelihood estimates of $\beta_{1}$ and each of the other parameters in the model. This is known as limited information maximum likelihood (LIML; see Section 18.5 in Davidson and MacKinnon^69^). Confidence intervals can be obtained by the assumption of asymptotic normality of the parameter estimates.

The term ‘limited information’ means that only limited information on the structure of the model is used. In particular, if there were multiple outcomes or risk factors, only parameters from a single equation are estimated. This is in contrast to full information maximum likelihood or three-stage least squares (3SLS),^70^ in which the whole system of equations in the model is estimated simultaneously. Full-system methods have the potential advantage of efficiency, but the disadvantage of inconsistent estimation if one or more of these equations are misspecified. However, in the context of Mendelian randomization with a single outcome and a single risk factor, there is no distinction.

LIML has been called the ‘maximum likelihood counterpart of 2SLS’^71(p. 227)^ and gives the same causal estimate as the 2SLS and ratio methods with a single IV. As with 2SLS, estimates are sensitive to heteroscedasticity and misspecification of the equations in the model. Use of LIML has been strongly discouraged by some, as LIML estimates do not have defined moments for any number of instruments.^54^ However, use has also been encouraged by others, especially with weak instruments, as the median of the distribution of the LIML estimator is close to unbiased even with weak instruments.^56(p. 209)^

The LIML estimate can be intuitively understood as the effect $\beta_{1}$ that minimizes the residual sum of squares from the regression of the component of the outcome not caused by the exposure, $\left(y_{i}-\beta_{1}x_{i}\right)$, on the IV(s). Informally, the LIML estimator is the causal parameter for which the component of the outcome due to confounding is as badly predicted by the IVs as possible.

### 5.2 Bayesian methods

Inferences from a likelihood model can also be obtained in a Bayesian framework.^72^ Here, we consider a slightly different likelihood model from equation (10) that has been discussed previously in the literature.^42^ For each individual *i*, we model the observed exposure and outcome as coming from a bivariate normal distribution for $(X_{i},Y_{i})T$ with mean $(\xi_{i},\eta_{i})T$ and variance-covariance matrix ∑. The mean of the exposure distribution $\xi_{i}$ is assumed to be a linear function of the instruments $z_{\mathrm{ik}},k=1,\ldots ,K$, and the mean of the outcome distribution $\eta_{i}$ is assumed to be a linear function of the mean exposure $\xi_{i}$^73^

(11) $$(X_{i}Y_{i})∼N_{2}((\xi_{i}\eta_{i}),\Sigma )\xi_{i}=\alpha_{0}+\sum_{k}\alpha_{k}z_{\mathrm{ik}}\eta_{i}=\beta_{0}+\beta_{1}\xi_{i}$$

This model is similar to that for the LIML method, except that the causal parameter $\beta_{1}$ represents the causal effect between the true means $\xi_{i}$ and $\eta_{i}$ rather than the measured values of outcome and exposure. The model can be estimated in a Markov chain Monte Carlo framework, such as that implemented in WinBUGS.^74^ The output is a posterior distribution, from which the posterior mean or median can be interpreted as a point estimate, and the 2.5th and 97.5th percentiles as a ‘95% confidence interval’. With vague priors, the posterior distribution is similar to the frequentist likelihood function.^42^

An advantage of the Bayesian approach is that, under the modelling assumptions, valid inferences can be obtained that are not based on asymptotic results, but rather on the posterior distribution of the causal parameter. Simulations have shown that frequentist properties of inference (such as coverage levels) are more robust in the Bayesian approach with weak instruments.^42^

### 5.3 Likelihood-based methods with binary outcomes

Maximum likelihood and Bayesian methods can be applied to binary outcomes. If we assume a linear model of association between the logit-transformed probability of an event ($\pi_{i}$) and the exposure (a logistic-linear model), and a Bernoulli distribution for the outcome event, as in the following model

(12) $$x_{i}∼N(\xi_{i},\sigma_{X}^{2})y_{i}∼\text{Bernoulli}(\pi_{i})\xi_{i}=\alpha_{0}+\sum_{k=1}^{K}\alpha_{k}z_{\mathrm{ik}}\text{logit}(\pi_{i})=\beta_{0}+\beta_{1}\xi_{i}+\beta_{2}(x_{i}-\xi_{i})$$

Estimates can be obtained by maximization of the joint likelihood, or in a Bayesian framework, obtaining posterior distributions by Markov chain Monte Carlo methods. Inclusion of the coefficient $\beta_{2}$ is similar to a control function approach (Section 4.3), in which the first-stage residual is adjusted for in the second-stage regression of a two-stage method. Alternatively, this term can be omitted, although formally this results in the likelihood model being misspecified.^42^ Simulations have suggested that both approaches give reasonable estimates, although they are affected by non-collapsibility.

Log-linear models can in principle be estimated in the same way, although care is needed to ensure that the probabilities $\pi_{i}$ do not exceed 1 at any point in the estimation procedure.

### 5.4 Comparison of two-stage and likelihood-based methods

In the two-stage methods, the two stages are performed sequentially. The output from the first-stage regression is fed into the second-stage regression with no acknowledgement of uncertainty. In the likelihood-based methods, the two stages are performed simultaneously: the *α* and *β* parameters are estimated at the same time. Uncertainty in the first-stage parameters is acknowledged and feedback between the regression stages is possible. The uncertainty in the estimate of the causal parameter *β*_1_ is therefore better represented in the likelihood-based approaches if there is non-negligible uncertainty in the first-stage regression model. This provides a heuristic justification why likelihood-based methods perform better than two-stage methods with weak instruments (see Section 7).

### 5.5 *k*-class estimators

The LIML and 2SLS estimators are part of a wider class of estimators known as *k*-class estimators.^75^ The *k*-class estimator of the causal effect *β*_1_ from equation (10) is

(13) β^k=[YT(I-kPZ)Y]-1[YT(I-kPZ)X]

where *Y* is a *N* by 1 matrix of the outcome, *Z* is a *N* by *K*+1 matrix of the IVs (including a constant term), and *X* is a *N* by 2 matrix of the risk factor and a constant term. The matrix $P_{Z}=I-Z(ZTZ)-1ZT$ is the projection matrix of *Z*.^76^ If *k*=1, then β^k is the 2SLS estimator. If *k* is the smallest root of $\det(YTY-kYTP_{z}Y)=0$, then β^k is the LIML estimator. Several other choices are available, including the bias-corrected 2SLS estimator,^54^ and Fuller’s *k* estimator.^77^ These estimators are beyond the scope of this review.

## 6 Semi-parametric methods

A semi-parametric model has both parametric and non-parametric components. Typically semi-parametric estimators with IVs assume a parametric form for the model relating the outcome and exposure, but make no assumption on the distribution of the errors. Semi-parametric models are designed to be more robust to model misspecification than fully parametric models.^78^

### 6.1 Generalized method of moments

The generalized method of moments (GMM) is a semi-parametric estimator designed as a more flexible form of 2SLS to deal with problems of heteroscedasticity of error distributions and non-linearity in the two-stage structural equations.^61,79^ With a single instrument, the estimator is chosen to give orthogonality between the instrument and the residuals from the second-stage regression. Using bold face to represent vectors, if we have

(14) E(Y|do(X=x))=f(x;β)

then the GMM estimate is the value of **β** such that

(15) $$\sum_{i}(y_{i}-f(y_{i};\beta ))=0\text{and}\sum_{i}z_{i}(y_{i}-f(y_{i};\beta ))=0$$

where the summation is across *i*, which indexes study participants. Equation (14) is a structural model, where E(Y|do(X=x)) is the expectation of the distribution that *Y* would take if we forced *X* to equal *x* for all individuals.^80^

In the linear (or additive) case, $f(y_{i};\beta )=\beta_{0}+\beta_{1}x_{i}$; in the log-linear (or multiplicative) case, $f(y_{i};\beta )=\text{expit}(\beta_{0}+\beta_{1}x_{i})$; and in the logistic case, $f(y_{i};\beta )=\text{expit}(\beta_{0}+\beta_{1}x_{i})$; where $\beta_{1}$ is our causal parameter of interest and expit(z)=(1-exp(-z))-1, the inverse of logit(*z*). We can solve the two equations in (15) numerically.^16^

GMM estimates are sensitive to the parameterization used. For example, estimates from the estimating equations (15) and from

(16) $$\sum_{i}(y_{i}\;f(y_{i};\beta )-1-1)=0\text{and}\sum_{i}z_{i}(y_{i}\;f(y_{i};\beta )-1-1)=0$$

may be different in finite samples, although they each assume the same structural model between the outcome and exposure. With a binary outcome, the second set of estimating equations is usually used, as this corresponds to a multiplicative error structure.^15^

When there is more than one IV, $z_{i}$ becomes $z_{\mathrm{ik}}$ and we have a separate estimating equation for each instrument *k*=1, … , *K*. The orthogonality conditions for each instrument cannot generally be simultaneously satisfied. The estimate is usually taken as the minimizer of the objective function

(17) (y-f(x;β))TZ(ZTΩZ)-1ZT(y-f(x;β))

where $\text{Z}=(1z_{1}\ldots z_{K})$ is the *N* by *K* + 1 matrix of instruments, including a column of 1s. Although this gives consistent estimation for general matrix Ω, efficient estimation is achieved when $\Omega_{\mathrm{ij}}=\text{cov}({ɛ}_{i},{ɛ}_{j})$ (*i*,*j* = 1, … , *N*), where ε_*i*_ is the residual $y_{i}-f(y_{i};\beta )$.^81^

As the estimation of Ω requires knowledge of the unknown **β**, a two-step approach is suggested. We firstly estimate β* using (ZTΩZ)=I, where *I* is the identity matrix, which gives consistent but not efficient estimation of **β**. We then use $e_{i}=y_{i}-f(y_{i};\beta *)$ to estimate $ZT\Omega Z=\sum_{i}z_{i}z_{i}T{ɛ}_{i}^{2}$ as $\sum_{i}z_{i}z_{i}Te_{i}^{2}$ in a second-stage estimation.^79^

### 6.2 Continuous updating estimator

An alternative approach is to express the weighting matrix Ω as a function of the causal parameter, and then to estimate the causal parameter and weighting matrix simultaneously by minimizing equation (17). This is known as the continuous updating estimator (CUE), as the weighting matrix updates during the estimation procedure.^82^ The CUE is similar to the LIML estimator in terms of confidence intervals and finite sample bias,^48^ and the two estimators are equivalent if the residuals *e*_*i*_ defined above are homoscedastic.^83^ However, the CUE does not make the homoscedasticity assumption. The CUE and two-step GMM estimators have the same asymptotic distribution.^83^

### 6.3 G-estimation of structural mean models

G-estimation of a structural mean model (SMM) is another semi-parametric estimation method^84,85^ designed in the context of randomized trials with incomplete compliance.^86,87^ The potential outcome *Y*(*x*) is defined as the outcome which would have been observed if the exposure *X* were set to *x*. In particular, the exposure-free outcome *Y*(0) is the outcome which would have been observed if we had set *X* to 0 rather than it taking its observed value of *x*.^78^

An explicit parametric form is assumed for the expected difference in potential outcomes between the outcome for the observed *X* = *x* and the potential outcome for *X*=0. In the continuous case, the linear or additive SMM is

(18) $$E(Y(x)-Y(0)|X=x,Z=z)=\beta_{1}x$$

and β_1_ is taken as the causal parameter of interest. In the context of non-compliance, this is referred to as the ‘effect of treatment on the treated’.^88^ In this equation, there is no effect modification on the additive scale by *Z*.

As the expected exposure-free outcome *E*(*Y*(0)|*X* = *x*,*Z* = *z*) is statistically independent of *Z*, the causal effect is estimated as the value of β_1_ which gives zero covariance between $E(Y(0)|X=x,Z=z)=E(Y(x)-\beta_{1}x|X=x,Z=z)$ and *Z*. The estimating equations are

(19) $$\sum_{i}(z_{\mathrm{ik}}-{\bar{z}}_{k})(y_{i}-\beta_{1}x_{i})=0k=1,\ldots ,K$$

where ${\bar{z}}_{k}=\frac{1}{N}\sum_{i}z_{\mathrm{ik}}$ and the summation is across *i*, which indexes study participants.

Where the model for the expected outcomes is non-linear, this is known as a generalized SMM. With a binary outcome, it is natural to use a log-linear (or multiplicative) SMM

(20) $$\text{log}E(Y(x)|X=x,Z=z)-\text{log}E(Y(0)|X=x,Z=z)=\beta_{1}x$$

In this case, there is no effect modification by *Z* on the multiplicative scale.

The linear (additive) and log-linear (multiplicative) GMM and SMM approaches outlined give rise to the same estimating equations, and so software for GMM estimation can also be used for the estimation of a SMM.^89^ In the case of a single IV, the linear GMM and SMM estimates coincide with the corresponding ratio estimate. With multiple IVs, the estimating equations are not guaranteed to have a unique solution, and so an objective function can be obtained and minimized in the same way as in the GMM approach. Efficient estimates can be obtained using the same two-step method outlined for the GMM approach in Section 6.1.^90^

Unfortunately, a logistic SMM cannot be defined in the same way, as the moment conditions in the estimating equations depend on unmeasured covariates due to the non-collapsibility of the odds ratio.^91^ This problem also arises in the GMM case,^15^ although GMM is an estimation method and so does not require the functional form relating the exposure and outcome to be given the interpretation of a structural model. For a SMM, the problem can be resolved by estimating *Y* assuming an observational model^90^

(21) $$\text{logit}E(Y|X=x,Z=z)=\beta_{0a}+\beta_{1a}x$$

where the subscripts *a* indicate associational, and substituting estimates of *Y* into the structural model

(22) $$\text{logit}E(Y(x)|X=x,Z=z)-\text{logit}E(Y(0)|X=x,Z=z)=\beta_{1c}x$$

where the subscript *c* indicates causal. The associational parameters can be estimated by logistic regression, leading to estimating equations

(23) ∑i(zik-z¯k)expit(Y^(x)-β1cxi)=0k=1,…,K

where Y^(x)=β^0a+β^1ax.

### 6.4 Potential lack of unique estimate with binary outcomes

An issue with semi-parametric IV estimation in practice is that an estimate of the causal parameter may not be uniquely determined in finite samples. A parameter in a statistical model is identified if its value can be uniquely determined from the joint distribution of the observed variables in an infinite sample.^92^ However, for a semi-parametric instrumental variable analysis with a single IV and a binary outcome, the estimating equations may be almost flat in the vicinity of the solution to the equations (known as weak identification) or there may be multiple or no parameter values which satisfy the estimating equations.^93^ This is especially likely if the IV is weak. Consequently, estimates and standard errors reported by automated commands for GMM or SMM estimation can be misleading.

It is recommended that applied investigators wanting to use a GMM or SMM approach should plot the objective function of the relevant estimating equations for a large range of values of the parameter of interest to check if there is a unique solution. If there is not, this should be reported as an indication that there is a lack of information on the parameter in the data. An alternative estimation technique can be used, such as a two-stage method, but identification will then rely on stronger assumptions.

A summary of features of the IV methods considered in this paper is given in Table 1. Having discussed various methods for IV estimation, we proceed to consider practical issues in the estimation of causal effects, in particular the use of weak instruments.

**Table 1.** Summary table of features of instrumental variable methods considered in this paper.

Ratio method	Can only be used with a single IV. Can be used with summarized data.
Two-stage methods	Efficient, but biased with weak instruments. Robust to misspecification in the first-stage model. Flexible to incorporate different exposure–outcome models.
Likelihood-based methods	Asymptotically efficient and less biased provided that the parametric assumptions hold. Requires coding by hand outside of the linear case.
Semi-parametric methods	Less efficient, less stringent assumptions. No guarantee of unique estimate in finite samples.

## 7 Weak instruments

A ‘weak instrument’ is defined as an IV for which the statistical evidence of association with the exposure is not strong. A weak IV differs from an invalid IV (one that does not satisfy the IV assumptions) in that the IV estimate using a weak IV is asymptotically unbiased; a weak IV can be made stronger by increasing the sample size. The F statistic in the regression of the exposure on the IV (also known as the Cragg–Donald F statistic^94^) is usually quoted as a measure of the strength of an instrument.^95^ IV estimates demonstrate systematic finite sample bias, typically in the direction of the observational (confounded) association between the exposure and outcome.^96^ IVs with an F statistic less than 10 are often labelled as ‘weak instruments’.^97^ The value was chosen as the bias of the 2SLS IV estimate with an IV having an expected F statistic value of 10 is limited to 10% of the bias of the observational association. Such a binary characterization of IVs as weak or strong is misleading, as the bias is a continuous phenomenon. Additionally, the expected magnitude of bias does not depend on the value of the F statistic for a particular dataset, but the expected value of the F statistic (also known as the concentration parameter^95^). The F statistic is highly variable between datasets generated under the same data-generating mechanism, and so the F statistic in a given dataset cannot be relied on as an accurate predictor of the expected bias in that dataset.^19^

### 7.1 Problems of estimates using weak instruments

Aside from the bias suffered by IV estimates with weak instruments, the distribution of such IV estimates differs substantially from a normal distribution. Confidence intervals that rely on the asymptotic normality of IV estimates (such as those typically reported by statistical software packages for 2SLS or LIML methods) tend to underestimate the true uncertainty in the estimates, leading to inflated Type I error rates. With large numbers of IVs (10 or more), standard confidence intervals from the LIML method with weak instruments are too narrow and a correction is needed (known as Bekker standard errors).^98,83^ Tables have been produced for the 2SLS method giving the expected value of the F statistic at which the maximum possible Type I error rate is less than 10%, 15%, 20%, and 25%.^99^ (Although the maximum error rate is only achieved when the correlation between the exposure and outcome is one, so such extremes should not be expected in practice.) The power of a study with weak instruments may also be low, although this is not necessarily the case if there are many weak instruments. Correlation between IV estimates and standard errors can lead to bias being accentuated in a meta-analysis of IV estimates, as in the sampling distributions, estimates closer to the observational association have greater estimated precision.^100^

These problems arise for IVs that are weak, but otherwise satisfy the IV assumptions. A more further problem with weak instruments is sensitivity to violations of the IV assumptions: while studies with stronger instruments can be insensitive to moderate violations of the IV assumptions, studies with weak instruments are extremely sensitive to tiny violations in the assumptions.^101^

### 7.2 Comparison of methods with single instrumental variable

As previously stated, if there is a single IV, the ratio, 2SLS, LIML, GMM and SMM IV estimates coincide. Although this IV estimate does not have a finite mean, simulations have shown that the median bias is close to zero even when the expected F statistic is around 5.^42^ An F statistic of 5 corresponds to a p-value for the association of the IV with the exposure of 0.03. It is unlikely that a variable would be used as an IV in practice unless the variable is expected to achieve a p-value of less than 0.03 in the sample under analysis. A causal effect and its direction can be assessed with a single IV by testing for an association between the IV and outcome; this association will be a valid test of the null hypothesis of no causal effect even with a weak instrument.^9^ Hence, unless bias is introduced via another mechanism (such as choosing between candidate IVs on the basis of their strength in the data under analysis^100^), the problems of weak instruments should not arise with a single instrumental variable.

### 7.3 Multiple instrument variables and score-based approach

The bias of the IV estimate from the 2SLS method with a continuous outcome is approximately 1/*E*(*F*) of the bias of the observational association, where *E*(*F*) is the expected value of the F statistic from the first-stage regression. In contrast, the median bias of estimates from the LIML and CUE methods is close to zero, making them useful tools for sensitivity analysis. However, in finite samples with multiple IVs, the LIML method is generally less efficient than 2SLS.^98^ A simulation analysis showed that confidence intervals from the LIML and CUE methods with large numbers of IVs were much wider than those from other IV methods, despite nominal coverage levels not being maintained^83^; use of Bekker standard errors to maintain nominal coverage levels resulted in confidence intervals that were wider still.

A recent innovation in Mendelian randomization is to reduce data from multiple instrumental variables into a single univariate score, known as an allele score or genetic risk score.^102^ An unweighted score is constructed as the total number of exposure-increasing alleles present in the genotype of an individual. A weighted score can also be considered, in which each IV contributes a weight reflecting the effect of the corresponding genetic variant on the exposure. If an individual *i* has *g*_*ik*_ copies of the exposure-increasing allele for each variant *k* = 1, … , *K*, then their unweighted score is $z_{i}=\sum_{k=1}^{K}g_{\mathrm{ik}}$. If all genetic variants are diallelic SNPs, this score takes integer values between 0 and 2*K*. If the weight for variant *k* is *w*_*k*_, then their weighted score is $z_{i}=\sum_{k=1}^{K}w_{k}g_{\mathrm{ik}}.$ Either score can then be used in an IV analysis.

If the weights in a weighted score are derived naively from the data under analysis, then the score-based approach is effectively a 2SLS analysis, and there is no methodological benefit of the approach to a 2SLS analysis in terms of bias. However, if the weights are determined externally from prior knowledge or an independent data source, or from the data under analysis by a cross-validation or jackknife approach,^103^ then effectively the IV analysis uses a single instrumental variable, reducing bias to negligible levels. Additionally, when the true effect sizes of the IVs do not vary greatly, score-based approaches have been shown to give greater precision than LIML and related methods, even when the weights for the score are misspecified or an unweighted score is used.^83,102^ We would therefore recommend a score-based approach to IV analysis when there are a multiple potentially weak IVs.

A caveat is that the use of large numbers of IVs increases the possibility that one or more of the IVs is invalid, leading to potentially misleading estimates.^101^ It is therefore important not only to cite genetic associations with the outcome for the allele score but also for the constituent genetic variants, so that the consistency of estimates across the genetic variants can be assessed. Although allele scores can address a wide range of analysis questions in Mendelian randomization, more complex questions (such as analyses with multiple risk factors^104^) cannot be addressed using an allele score method and require the use of alternative methods.

## 8 Discussion

In this final section, we discuss various practical topics relevant to IV estimation, as well as extensions to the scenario considered in this paper of individual-level data from a single source to estimate a linear causal effect.

### 8.1 Non-linear relationships

With a linear exposure–outcome relationship, the estimate from a linear IV analysis, such as using the ratio or 2SLS method, is the average change in the outcome for a uniform change (usually a 1 unit increase) in the distribution of the exposure across the population for all individuals whose exposure level is influenced by their value of the IV.^27^ For a non-linear relationship, a linear IV estimate approximates the same population-averaged causal effect when the change in the distribution of the exposure associated with the IV is small, and when the linear IV estimate is scaled to represent the effect of a change in the exposure of similar magnitude to that associated with a change in the IV.^34^

Although non-linear parametric two-stage IV methods have been proposed, inferences based on such methods have been shown to be highly sensitive to the choice of parameterization.^105,106^ Equally, non-parametric two-stage IV methods have been explored,^107^ but evidence on the shape of the causal relationship can only be inferred for the range of fitted values for the exposure (X^|*Z*). A recent development is the estimation of local IV estimates within strata of the exposure.^34^ However, if the exposure is stratified on directly, misleading results may be obtained. This is because the exposure lies on the causal pathway between the IV and the outcome, and so conditioning on the exposure induces an association between the IV and confounders. This can be circumvented by initially subtracting the effect of the IVs on the exposure from the exposure measurement, to obtain the ‘IV-free exposure’. This quantity, representing the expected value of the exposure for an individual if their IVs took the value zero, can then be safely conditioned on.^108^ Conditioning on this quantity is equivalent to conditioning on the residual from the first-stage regression,^65^ known as the ‘control function approach’.^109^ For this non-linear estimation approach to be valid, it is necessary for the association of the IV with the exposure in the population to remain constant at different levels of the exposure.^110^

### 8.2 Use of measured covariates

If we can find measured covariates which explain variation in the exposure or a continuous outcome, then we can incorporate such covariates into our analysis. Including covariates generally increases efficiency and hence the precision of the causal estimate. However, it may lead to bias in the causal estimate if a covariate is on the causal pathway between exposure and outcome or is a collider or causally downstream of a collider. A collider is a variable that is influenced both by the exposure or a causal consequent thereof and by the outcome or a causal consequent thereof.^111^ There may also be bias if the analysis model including the covariate is misspecified. In a two-stage estimation, any covariate adjusted for in the first-stage regression should also be adjusted for in the second-stage regression^112^; failure to do so can cause associations between the IV and confounders leading to bias.^56(p. 189)^

In some cases, adjustment for covariates is necessary to ensure validity of the IVs, as the IV assumptions only hold conditional on the covariates. An example in Mendelian randomization is the case of population stratification, in which the sample population consists of subpopulations that have different distributions of the IV and outcome.^5^ These subpopulations may correspond to ethnic subgroups in the population. An association between the IV and outcome may solely correspond to differences in ethnicity and not to any biological effect of the exposure. This can be addressed at least partially with genome-wide data, by adjusting for genetic principal components in the IV analysis,^113^ although there is no theoretical guarantee that adjustment for genetic principal components will resolve the problem of population stratification. In general, aside from these cases, we discourage adjustment for covariates in the primary analysis result for the assessment of a causal effect.

### 8.3 Overidentification tests

When more than one instrument is used, an overidentification test, such as the Sargan test,^114^ can be carried out to test whether the associations of the instruments with the outcome are statistically compatible with a single linear causal effect of the exposure. Overidentification means that the number of instruments used is greater than the number of exposures measured. (The latter is usually one, although analyses with multiple exposure variables have also been considered.^115,116^) When there is more than one IV for a single exposure, separate causal estimates can be calculated using each IV in turn. The overidentification test assesses whether the parameters being estimated by each IV separately are the same, or equivalently whether the IVs have residual associations with the outcome once the main effect of the exposure has been removed.^117^ Failure of an overidentification test indicates heterogeneity in the effect estimates from each IV, which suggests that the IV assumptions may be violated for one or more genetic variants.

Overidentification tests are omnibus tests, where the alternative hypothesis includes failure of IV assumptions for one IV, failure for all IVs, a non-linear association between the exposure and outcome, and that different IVs identify different magnitudes of causal effect.^118^ However, in many practical cases they have low power,^119^ and so failure to reject an overidentification test should not be overinterpreted as positive evidence for the validity of the IV assumptions.

### 8.4 Endogeneity tests

Some applied Mendelian randomization analyses have reported on whether there is a difference between the observational and IV estimates as the primary outcome of interest^120^; this can be formally tested using the Durbin–Wu–Hausman test.^118^ This is a test of equality of the observational and IV estimates, where a significant result indicates disagreement between the two estimates. It is called an endogeneity test.

While an informal comparison of the observational and causal estimates may be reasonable, for instance to judge whether a Mendelian randomization investigation has sufficient power for a null result to be informative, an endogeneity test should not be viewed as a primary analysis result. This is because the outcome of an endogeneity test neither implies the presence nor the absence of a causal effect. It is more appropriate to consider the presence or absence of a causal effect as the subject of investigation.^39^ The confidence interval of the causal estimate gives the researcher a sense of the plausible size of any possible causal effect based on the data available.

### 8.5 Power of IV analyses

The confidence interval of an IV estimate is typically much wider compared with that of an observational estimate, for example from a regression analysis. The sample size required to obtain precise enough causal estimates to be clinically relevant can be very large.^121^ A rule of thumb for power is that the sample size for a conventional analysis should be divided by the coefficient of determination (*R*^2^) of the IV on the exposure.^122^ Formulae for power and sample size calculation have been published with a continuous outcome^123,124^ and with a binary outcome,^125^ and online tools for performing these calculations are available (http://glimmer.rstudio.com/kn3in/mRnd/ and http://sb452.shinyapps.io/power/).

### 8.6 Summarized data

In an applied context, it may not be practical or desirable to share individual-level data on the IV, exposure, and outcome. With a single IV, the ratio method can be implemented simply using summarized estimates on the associations of the IV with the outcome and with the exposure. Methods for obtaining IV estimates using summarized data on multiple IVs have been discussed elsewhere^126^: these include a likelihood-based method for the summarized association estimates, and an inverse-variance weighted method combining the ratio estimates from each IV in a meta-analysis model.^127^ A tool to implement these methods is available online (http://sb452.shinyapps.io/summarized/).

### 8.7 Subsample and two-sample Mendelian randomization

Especially when the outcome is binary, the sample size required in a Mendelian randomization experiment may be prohibitively large due to the expense of collecting data on the exposure. In this case, a subsample IV approach may be a cost-effective approach. Rather than collecting data on the exposure from the entire study sample, exposure data can be measured for a random subsample of (control) participants. As the association between the IV and the exposure is typically stronger than that between the IV and the outcome, the precision of the IV estimate may not be noticeably affected by reducing the sample size on which the exposure is measured. Simulations have shown that a subsample IV analysis with exposure data on only 10% of participants may retain 90% of the power of the full-sample IV analysis.^128^ Estimates and confidence intervals with a single IV can be calculated using the ratio method and Fieller’s theorem (Section 3.4). With multiple IVs, a modified version of the two-stage least squares method can be used.^129^

An alternative design is two-sample Mendelian randomization, in which the associations between the IV(s) and the exposure and between the IV(s) and the outcome are estimated from non-overlapping sets of individuals.^130^ Although this may simply reflect the absence of a dataset with concomitant information on all three of the IV(s), exposure, and outcome, it is also a potentially efficient design strategy for Mendelian randomization, particularly in view of the increasing public availability of summarized data on genetic associations with risk factors and disease outcomes from large consortia. Several consortia with large numbers of participants, such as CARDIoGRAMplusC4D for coronary artery disease^131^ and DIAGRAM for type 2 diabetes,^132^ have published summarized data on the association of catalogues of genetic variants with either risk factors or disease status. These provide precise estimates of genetic associations which can be used to obtain causal estimates, provided the genetic variants included in the analysis are restricted to those for which the IV assumptions are valid. This makes two-sample Mendelian randomization using summarized data a worthwhile analysis strategy from the perspective of power, and one that should be encouraged in practice, particularly if there is available summarized data on genetic associations with the outcome.^133^ The only caveat is that the two samples used should be as similar as possible (particularly with respect to nationality/ethnicity, but also to other population characteristics). This is because genetic variants that are valid IVs in one population may not be valid IVs in another population, and to ensure that the magnitudes of genetic associations with the risk factor in the first sample are relevant estimates of the genetic associations in the second sample.

### 8.8 Meta-analysis

The demands of power often demand synthesis of evidence from multiple, possibly heterogeneous studies. Data from multiple studies can be combined to provide a single causal estimate in a meta-analysis model. If it is possible to estimate the causal effect in each study, meta-analysis can be performed directly on these estimated causal effects, for example using inverse-variance weighting.^134^ However, a study-level meta-analysis can accentuate weak instrument bias,^100^ and so it is preferable to combine either individual-level or summarized data from each study. Additionally, it may be that some studies only provide data on one of the exposure or the outcome, and so a causal effect cannot be estimated from that study alone. A hierarchical model can be therefore proposed, in which the study-specific parameters are pooled in a between-study model. This could also be a more efficient use of data if the same genetic variants were used in separate studies. Such a model can be fitted using likelihood methods on either individual-level data or summarized data from each study.^135^

### 8.9 Robust methods

Instrumental variable estimation methods that are robust to the violation of the IV assumptions for some or all of the ‘instruments’ have been the subject of recent methodological development. A full exposition of these methods is beyond the scope of this manuscript, but we provide a brief overview of some of these developments.

Given multiple candidate IVs, if over half of the ‘instruments’ satisfy the IV assumptions, then the median of the IV estimates using each of the ‘instruments’ individually is a consistent estimate of the causal effect.^136^ A more efficient estimator than this simple median can be constructed by a weighted median method.^137^ Here, rather than assuming over 50% of the ‘instruments’ are valid IVs, we assume that ‘instruments’ representing over 50% of the weight are valid IVs.

Alternatively, a consistent causal estimate can be obtained by allowing the ‘instruments’ to have direct effects on the outcome, but limiting the total magnitude of these direct effects, for example using L1-penalization.^138^ Confidence intervals under the assumption that over 50% of the ‘instruments’ are valid IVs have also been developed.^139^ Software for performing this penalization method is available in the R package sisVIVE.^140^

Finally, direct effects of the IVs on the outcome can be accounted for by regressing the IV – outcome association estimates on the IV – exposure association estimates. If the associations of IVs with the outcome consist of a causal component, representing the effect of the exposure on the outcome, and a pleiotropic component, representing the direct effect of the IV on the outcome not via the risk factor, then the intercept from this regression model is an estimate of the average direct effect of each IV on the outcome.^141^ If the IV assumptions are satisfied, then the true direct effect of each IV on the outcome is zero. A test of whether the intercept estimate differs from zero is an assessment of the presence of directional (that is, unbalanced) pleiotropy. This is equivalent to Egger’s test, an assessment of small sample bias (including publication bias) in meta-analysis.^142,143^

Under a weaker set of assumptions than the standard IV assumptions (namely that the direct effects of IVs on the outcome are distributed independently of instrument strength^144^), the slope parameter from this regression with an intercept term is a consistent estimate of the causal effect. This method can be used to give a consistent estimate of the causal effect even if all the ‘instruments’ are invalid, but it requires the standard untestable assumption of no direct effects of the IVs on the outcome to be replaced with another untestable (albeit weaker) assumption.

### 8.10 Summary

Methods for IV analysis range from the very simple to the more complicated. The development of complex methods has been driven by the desire to produce efficient estimates, for example by integrating data on multiple IVs or multiple studies, or to provide robustness against weak instruments or misspecification of the modelling assumptions.

In conclusion, we emphasize that the main difficulty in an IV analysis is not the choice of analysis method but rather the choice of IVs and the assessment of the IV assumptions. If the IV assumptions do not hold, then inferences from any analysis method will be unreliable.^26^ Although there are some contexts where a particular method is unsuitable (such as with large numbers of weak instruments), there is no single universal ‘best’ IV estimation method. Instead, the use of different IV methods provides sensitivity analyses to assess whether the estimate given by a particular choice of method is credible.
